# A Study of the Curing and Flammability Properties of Bisphenol A Epoxy Diacrylate Resin Utilizing a Novel Flame Retardant Monomer, bis[di-acryloyloxyethyl]-*p*-*tert*-butyl-phenyl Phosphate

**DOI:** 10.3390/ma10020202

**Published:** 2017-02-20

**Authors:** Syang-Peng Rwei, Yu-Ming Chen, Whe-Yi Chiang, Yi-Tien Ting

**Affiliations:** Institute of Organic and Polymeric Materials, National Taipei University of Technology, #1, Sec 3, Chung-Hsiao E. Rd, Taipei 10608, Taiwan; t7350304@gmail.com (Y.-M.C.); wheyichiang@gmail.com (W.-Y.C.); jerry936314@gmail.com (Y.-T.T.)

**Keywords:** DAPP (bis[di-acryloyloxyethyl]-*p*-*tert*-butyl-phenyl phosphate), BPDCP (4-*tert*-butylphenyl dichlorophosphate), kinetics of pyrolysis

## Abstract

A UV-curable, flame-retardant monomer, DAPP (bis[di-acryloyloxyethyl]-*p*-*tert*-butyl-phenyl-phosphate), was synthesized based on BPDCP (4-*tert*-butylphenyl-dichloro phosphate) and HEA (2-hydroxy ethyl acrylate). DAPP was blended with regular bisphenol A epoxy acrylate (BAEA) in various ratios to yield various phosphorus contents. The TGA-IR (thermogravimetric analyzer interface with an infrared spectrometer) results demonstrate that compounding 30 mol % DAPP with BAEA significantly reduced the amount of released CO gas. In contrast, the peak intensity of CO_2_ is independent of phosphorus content. The limiting oxygen index (LOI), reaching the saturated value of 26, and the heat release rate (HRR) measured using a cone-calorimeter, 156.43 KW/m^2^, confirm the saturation point when 30 mol % DAPP was compounded into BAEA. A study of the kinetics of pyrolysis reveals that E_a_ decreases as the phosphorus content increases. Both the TGA-IR and pyrolysis results reveal that the phosphorus compound DAPP is easily decomposed during the initial stage of burning to form an insulating layer, which inhibits further burning of the resin and the consequent release of other flammable gases.

## 1. Introduction 

A non-halogen, flame-resistant coating can generally be produced by physically compounding phosphorus-containing additives into a polymeric matrix [1,2]. However, high additive loading and poor dispersion cause a coating solution to have an extremely high viscosity, resulting in difficulties in the curing process and unsatisfactory performance. Therefore, phosphorus-containing acrylate has recently attracted considerable attention as a flame-retardant oligomer for UV-cured films or coatings, owing to its non-halogenic characteristics and good dispersion of phosphorus throughout the compound [3,4,5,6,7]. These phosphorus-containing compounds have low toxicity, no dioxin generation, and no smoke during combustion. UV-curable reactive flame retardants have well-distributed domains on a molecular scale, and thus only a low concentration of the flame retardants is required to provide effective flame retardancy. 

A series of multifunctional oligomers/monomers have been reported as flame retardants for use in UV-curable systems [8,9,10]. Poly(biphenyl acryloxyethyl phosphate) has been blended with urethane acrylate EB 200 in various blend ratios to obtain UV-curable resins with effective flame retardancy [11]. A novel phosphorus monomer, 2,2-dimethyl-1,3-propanediol acryloyloxyethyl phosphate (DPHA), has been synthesized and blended with triglycidyl isocyanurate acrylate (TGICA) at various blend ratios to demonstrate the synergistic effects of these two compounds in a UV-curable acrylate system [12]. A melamine-based hyperbranched polyphosphonate acrylate (MHPA) has been synthesized and blended with the epoxy acrylate (EA) [13]. It has been claimed that adding 40 wt % MHPA can increase the LOI (Limiting Oxygen Index) of EA to 27. All the phosphorus-containing reactants were linear monomers that incorporate no aromatic moiety. On the other hand, the effect of a newly synthesized phosphorus monomer-containing aromatic nucleus on the flame retardancy of epoxy resin is reported here.

In this work, a novel flame-retardant monomer, DAPP (bis[di-acryloyloxyethyl]-*p*-*tert-*butyl-phenyl phosphate), based on BPDCP (4-*tert*-butylphenyl dichlorophosphate) and HEA (2-hydroxy ethyl acrylate), was designed and synthesized. A series of BAEA (bisphenol A epoxy acrylate) epoxies, with various phosphorus contents, were derived by blending with DAPP at various blend ratios. The UV curing kinetics of BAEA epoxies were investigated using photo-DSC. The combustion of the compounds was characterized using a cone calorimeter. Thermal degradation was characterized using TGA and monitored over time using TGA-IR (thermogravimetric analyzer interface with an infrared spectrometer).

## 2. Experimental

### 2.1. Materials

Both the bisphenol A epoxy acrylate resin (BAEA; chemical nomenclature: 4,4’-(1-Methylethylidene) bisphenol polymerized with 2-(chloromethyl)oxirane 2-propenoate)) and the phosphorus-containing compound (BPDCP) were purchased from Double-bond Chemical Co. (New Taipei City, Taiwan). The HEA was purchased from Aldrich Co. (St. Louis, MO, USA). 2-Hydroxy-2-methyl-1-phenyl-1-propanone (Darocur1173), purchased from Aldrich Co., was used as a photoinitiator. BPDCP was washed three times using toluene to eliminate soluble residual monomers or oligomers; other reagents were used as received.

The DAPP was synthesized as follows. Fifty grams of HEA were placed in a 500-mL flask containing 300 mL of toluene. A small amount of hydroquinone (HQ) and triethylamine (TEA) were then added to the flask as a reaction inhibitor and a condensated HCl trapper, respectively. The BPDCP in a mole ratio of BPDCP/HEA being 1:1 was slowly dropped into the reactor at 50 °C under a N_2_ atmosphere. The reactor was heated to 85 °C and kept at 85 °C for six hours until the OH peak in the FTIR spectrum of the mixture completely disappeared. The filtration was then performed at room temperature to collect the TEA sediments. Deionized water was mixed with the solution to remove the dissolved salt in the toluene. After getting rid of the water, the solution was then purified by distillation at 70 °C and 3 mmHg. Finally, a brown liquid in a yield of 77% was obtained.

### 2.2. Measurements

The UV curing kinetics was examined using a Perkin-Elmer PDSC (PhotoDSC; Doublebeam Photocalometric Accessory (DPA-7), Perkin Elmer Inc., Shelton, CT, USA) with wavelengths between 250 nm and 600 nm. The intensity of a UV lamp is 2 W/cm^2^. Since the intensity of the light source varies with age and other variables, the DPA 7 includes a photofeedback control loop to maintain constant lamp intensity. All kinetic analyses were based on the exothermic peak that was obtained from the PDSC curve [14,15,16,17]. Various amounts of the photoinitiator (Darocur 1173) were blended with pure DAPP in the absence of a solvent. Approximately 10 mg of the prepared sample was dropped into the DSC aluminum pan. The PDSC measurement was then made at 25 °C. The light intensity was the same for all experiments.

The dynamic mechanical analysis of the copolymers was investigated by DMA (PERKIN 7e, Perkin Elmer Inc., Shelton, CT, USA). The specimens had a length, width, and thickness of 12, 5, and 1 mm, respectively. The temperature was varied from 20 to 150 °C at a scan rate of 5 °C/min. The static force was kept at 70 mN; the dynamic force was set to 60 mN. The frequency used was 1 Hz and the amplitude was 5 μm. The test sample was cured by UV irradiation with intensity of 50 mW/cm^2^ for 10 min and then post-cured at 100 °C for 10 min before the DMA measurement.

The thermal degradation and flame-retardant performance of UV-cured film were investigated using TGA (Thermogravimetric Analysis, Perkin Elmer Pyris 1, Perkin Elmer Inc., Shelton, CT, USA), TG-IR (Perkin Elmer Pyris 1 TGA & Spectrum One FT-IR) and a cone calorimeter (Cone II, Atlas Electric Co., Spokane, WA, USA). TGA was carried out in a nitrogen atmosphere; a heating rate of 10 °C/min was used for the thermal degradation study and a rate of 10 to 40 °C/min was used in the investigation of the kinetics of pyrolysis. About 10 mg of UV cured sample was put in an aluminum crucible and heated from 25 to 700 °C. In the TG-IR measurement, the interface provided by Perkin Elmer connecting the TGA and IR spectrophotometer was maintained at 220 °C. The IR chamber was maintained at 200 °C and the spectrum was recorded every 6 s. 

The cone calorimeter is an apparatus for precisely determining the flammability of an organic material [18,19]. The measurement of the heat release rate is based on Huggett’s [20] principle that the gross heat of combustion of any organic material is directly related to the amount of oxygen that its combustion consumes (13.1 kJ/kg O_2_). A procedure that was adapted from ISO 5660-1 was carried out [21]. A test sample, encased in aluminum foil (100 × 100 × 2 mm^3^), was placed in the testing chamber and heated using a conical radiator with a power of 50kW/m^2^ for five minutes. The sample was wrapped in aluminum foil so that only the top surface was irradiated by the heat from the conical heater. The sides of the sample were not exposed to the radiative heat. The cone calorimeter software calculated the heat release rate (HRR) under radiation and other important factors that affected it.

LOI, the most frequently used index of the flammability of an organic material, is defined as the minimum percentage of oxygen in an oxygen–nitrogen mixture that can sustain the combustion of a specimen following ignition. The LOI measurements were made herein using a Santon Redcroft flame meter, modified by the method that was described by Nair et al. [22]. Powdered samples (500 mg) were placed in a ceramic cup (diameter 40 mm × height 4 mm) in the middle of an Atlas cylindrical chamber (diameter 80 mm × 200 mm) through the top of which a flame was inserted for 10 s. Nitrogen and oxygen gas mixture with various ratios of N_2_/O_2_ was set to flow at 12 L/min. The percent concentration of oxygen in the injected gas to support combustion for just 30 s was taken as the LOI. The advantage of this method for obtaining the LOI is that up to nine-tenths of the testing material that would be consumed by regular ASTM measurement can be spared. The standard deviation of the LOI was approximately 5%.

## 3. Results and Discussion

### 3.1. Confirmation of Synthesis

**Scheme 1:**
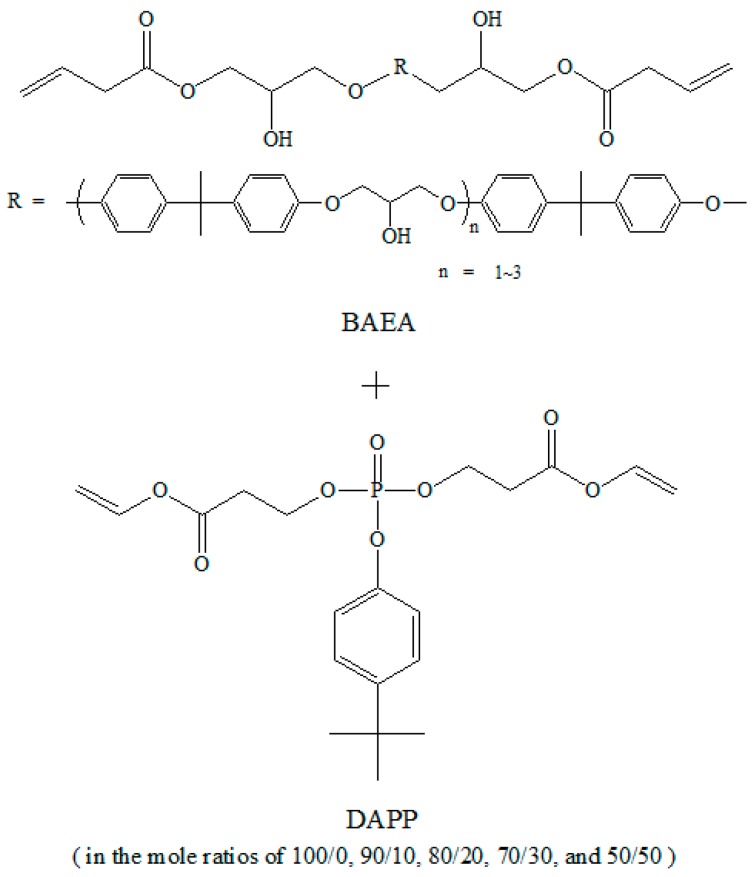
Reaction route of DAPP monomer and BAEA/DAPP polymer.

**Figure 1:**
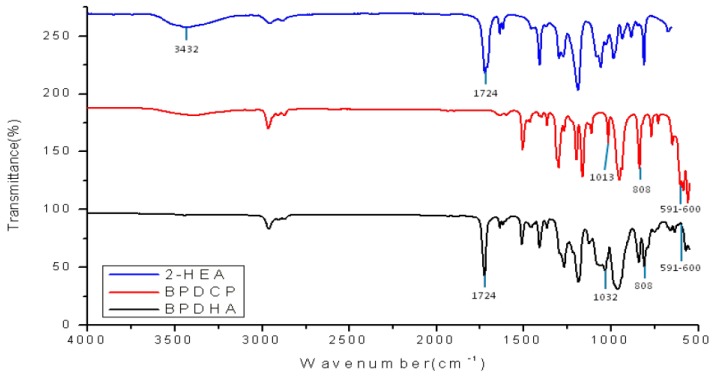
The FT-IR spectra of HEA, BPDCP, and DAPP.

In Scheme 1, DAPP was produced by reacting BPDCP with HEA. Figure 1 presents the FTIR spectra of species HEA, BPDCP, and DAPP. Figure 1 shows that the peaks associated with the OH group of HEA at 3300 cm^−1^ and the P–Cl group of BPDCP at 581–600 cm^−1^ had disappeared after the condensation reaction. For the newly synthesized compound, the P–O–C peak of BPDCP at 1013 cm^−1^ was shifted to 1032 cm^−1^, reconfirming the DAPP synthesis.

**Figure 2:**
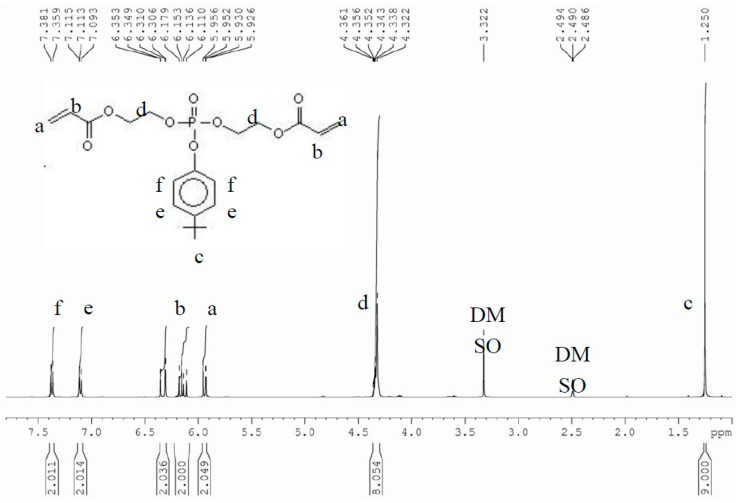
^1^H NMR spectrum of DAPP compound.

**Figure 3:**
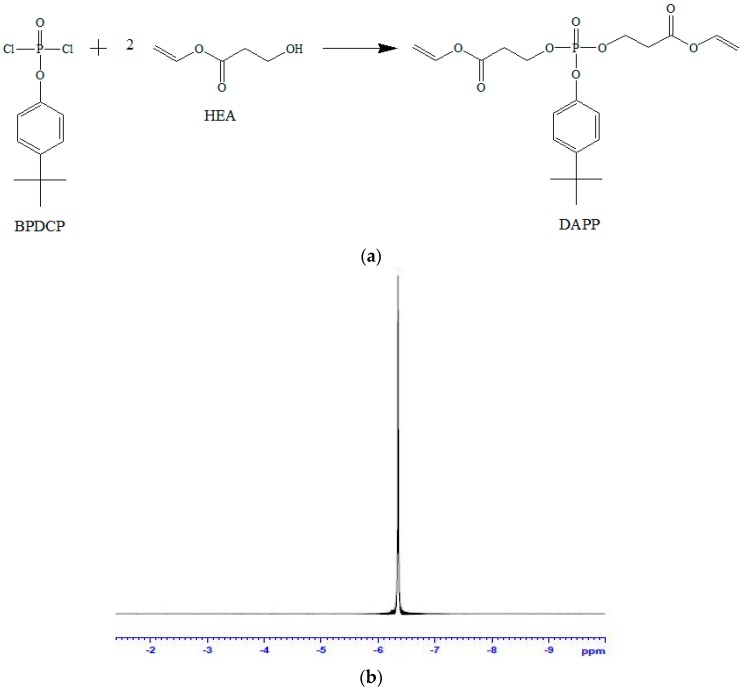
(**a**) Chemical structure; and (**b**) ^31^P NMR spectrum of DAPP compound.

The synthesized DAPP was further characterized by ^1^H NMR spectroscopy (Figure 2). Figure 3a shows the reaction of BPDCP and HEA to obtain DAPP.

The peaks in Figure 2 are assigned as follows; δ = 1.25 ppm (C(CH_3_)_3_), δ = 4.32–4.36 ppm (CH_2_CH_2_), δ = 7.09–7.11 and 7.35–7.38 ppm (aromatic proton), δ = 6.10–6.17 ppm (CH=CH_2_, trans), δ = 5.92–5.95 ppm (CH=CH_2_, cis), δ = 6.30–6.35 ppm (CH=CH_2_). Furthermore, the ^31^P NMR spectrum shows a single phosphorus resonance occurs at δ = −6.3–(−6.6) ppm (Figure 3b). All of the NMR and IR results confirm the successful synthesis of DAPP. 

### 3.2. UV Curing Kinetics

The autocatalyzed reaction model (Equation (1)), which is often utilized to describe the curing reaction, was used herein to analyze the UV-curing kinetics for various photoinitiator concentrations, acrylate sites, and reaction temperatures [23]:

dα/d*t* = *k* × α*^m^* × (1 − α)*^n^* (1)

where *m* is the order of the catalyzed reaction; *n* is the order of the catalyzed reaction; *k* is the rate constant of the reaction; and α is the reaction conversion ratio at a given temperature.

**Figure 4:**
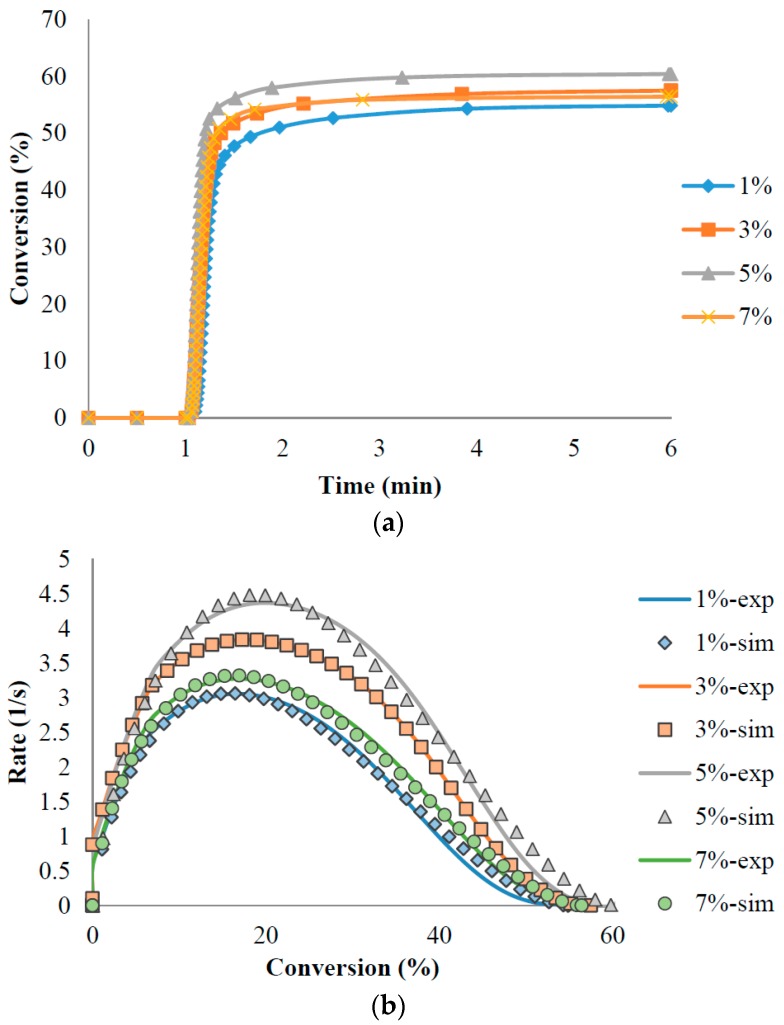
(**a**)The conversion rate as a function of time; (**b**) the reaction rate as a function of conversion for DAPP with different amounts of the photoinitiator.

Figure 4a,b show plots of the conversion ratio versus time and the reaction rate versus conversion ratio, respectively, for the pure DAPP system with various concentrations of photoinitiator. The simulated results are plotted as solid lines in Figure 4b. Table 1 shows the values of all parameters obtained by fitting. The simulated results fitted the experimental data very closely, revealing that the autocatalysis model effectively describes the UV curing mechanism of the system [24,25,26]. Table 1 shows that the optimal concentration of the photoinitiator is approximately 5 wt %. At this concentration, both the reaction rate constant *k* and the final conversion α are maximal. As the amount of photoinitiator increases, the concentration of free radicals increases and thus the probability of a reaction increases. The curing rate and the curing conversion are thereby increased. Once the initiator concentration reaches a threshold, some radicals collide with living polymers and act as terminators of the reaction; the curing rate and conversion no longer increase, and may even decrease.

UV curing does not only depend on collisions between pairs of acrylate groups, as in typical reaction kinetics. Immediately following the collision, a free radical must open the C=C double bonds of the acrylate groups, as discussed in our previous work [17]. However, all factors associated with the initiation stage or the propagation period of free radicals are incorporated into the rate constant *k* in Equation (1). Table 1 therefore demonstrates the reaction parameters, *k*, *m*, and *n*; only *k* is sensitive to the concentration of the initiator, while *m* and *n* are quite insensitive thereto.

### 3.3. Thermal Effects and Characterization of Pyrolysis

**Figure 5:**
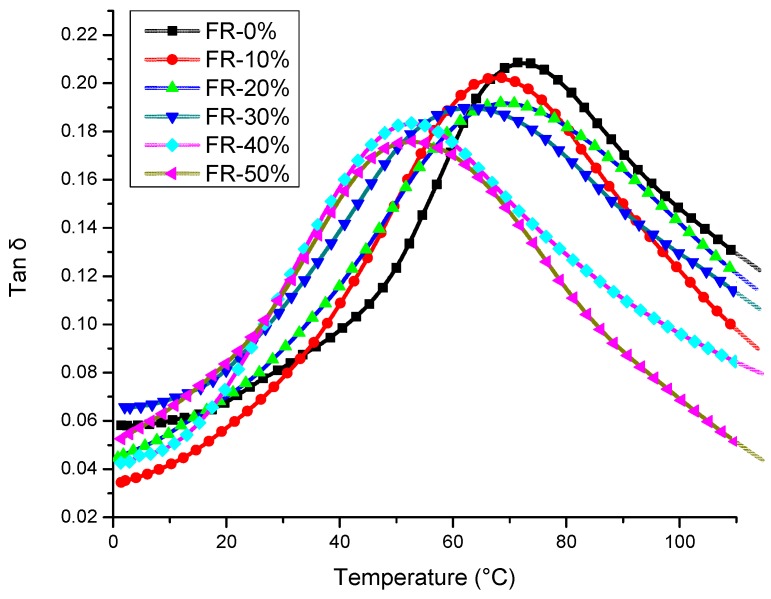
DMA curves of the cured films of DAPP/BAEA with various ratios.

Figure 5 plots tanδ against temperature, determined by DMA, for the BAEA/DAPP systems with molar ratios of 100/0, 90/10, 80/20, 70/30, 60/40, and 50/50. The tanδ spectrum reveals a major relaxation peak that corresponds to the transition of cured acrylates at *T_g_*. Table 2 shows *T_g_*, obtained from Figure 5. *T_g_* decreased as the DAPP content increased. DAPP is much more flexible than BAEA because DAPP has no rigid aromatic moiety, which is, on the contrary, present in the main chain structure of BAEA. Moreover, the phosphorus-containing main chain is more flexible than a pure carbon backbone. Incorporating such a phosphorus-containing monomer into the cured BAEA will therefore yield a cured acrylate with reduced chain rigidity, increased chain mobility, and weaker glass transition (*T_g_*).

**Figure 6:**
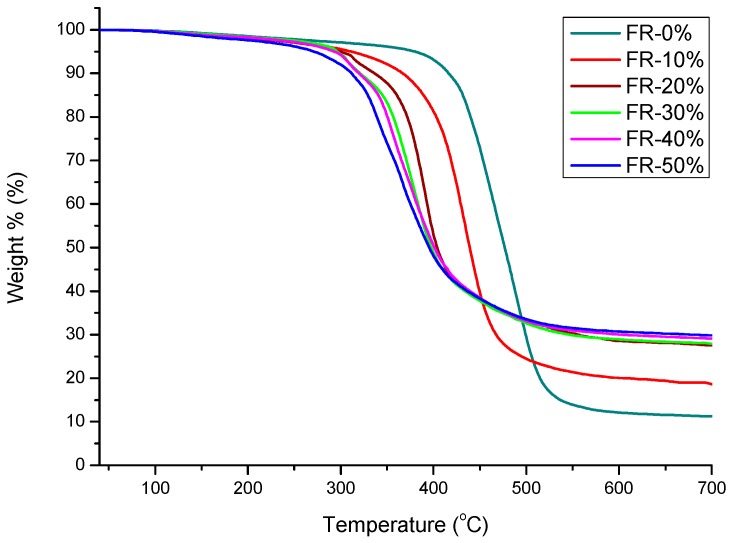
Thermogravimetric analysis (TGA) curves of the cured films of DAPP/BAEA with various ratios in nitrogen atmosphere.

The most common means of evaluating the thermal stability of cured material is thermogravimetric analysis (TGA). Figure 6 presents typical TGA thermograms of BAEA/DAPP systems with various composition ratios. The decomposition temperature *T_d_*, temperature at 5 wt % loss, and the residual mass percentage, called the char yield, of the cured films of DAPP/BAEA determined from the TGA results are shown in Table 2. The TGA results were obtained at a heating rate of 10 K/min. Table 2 firstly reveals that the *T_d_* value decreases as the DAPP content increases. In the early stage of decomposition, dehydration of the phosphorus-containing polymer is easily initiated, producing a layer of phosphate groups on the polymer surface that insulates the underlying polymer from heat and oxygen [27]. Moreover, the vapor released during dehydration may dilute the flammable gases and extinguish the flame. Accordingly, the decomposition temperature *T_d_* declined, as the phosphorus content increased. 

Table 2 secondly reveals that the char yield increased with the phosphorus content, reaching saturation at the BAEA/DAPP ratio between 70/30 and 60/40, suggesting that more than 30 mol % of the flame-retardant monomer should be added. The relationship between char yield and flame retardancy was reported and it indicated that the char yield following pyrolysis was linearly proportional to the oxygen index (LOI) [28]. Increasing the char yield can reduce the amount of generated combustion gases, the heat emitted by the pyrolysis reaction, the thermal conductivity of bulk material, and the flammability of polymers. The amount of oxygen required to ignite polymeric materials with a higher char yield is therefore significantly higher. Briefly, the formed intumescent char layer acts as a physical barrier that enhances thermal-oxidative stability by slowing down the transfer rates of heat and mass between the gas and condensed phases. Table 2 indeed reveals that the LOI reaches the saturated value of 26 at 30 mol % DAPP, and this value exceeds that of pure BAEA, which is 22.

**Figure 7:**
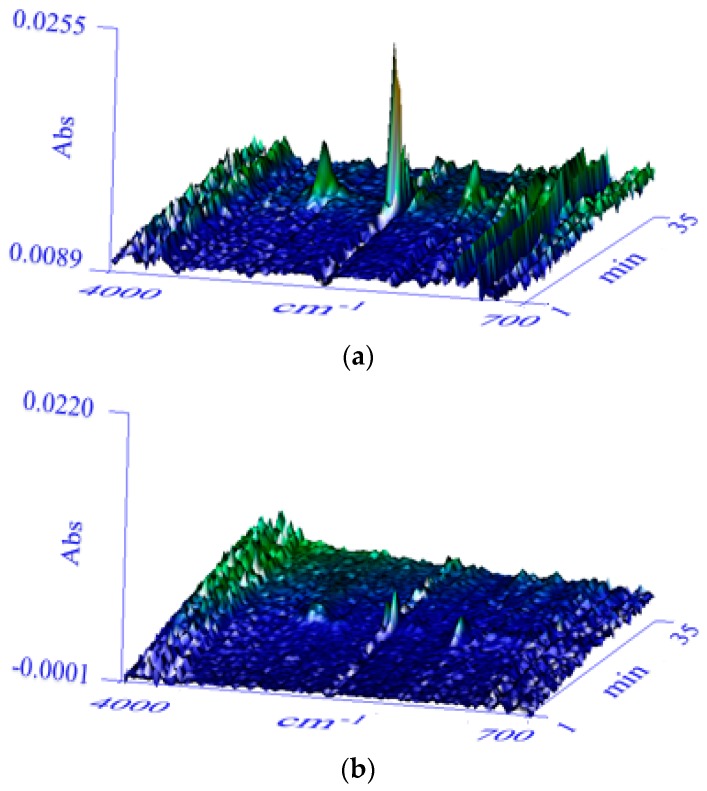
The 3D surface graph for the FTIR spectra of the evolved gases produced by (**a**) BAEA and (**b**) BAEA/DAPP 50/50, respectively, in a N_2_-rich environment pyrolysis.

The mode of thermal degradation can be elucidated using the TGA-IR technique that identifies the instantaneous evolution of volatilized products in the reaction. The effect of a phosphorus-containing compound on the decomposition of BAEA/DAPP polymer samples was investigated using a TGA-IR apparatus. The decomposition evolution was shown in Figure 7a,b which plot the amount of volatilized products that have escaped vs. time. The figures show the 3-D TGA-IR spectra of the chemical components of gaseous decomposed BAEA and BAEA/DAPP 50/50, respectively, in an N_2_-rich environment. Some characteristic absorption-peaks, including those of CO (2174 cm^−^^1^), CO_2_ (2304 cm^−^^1^), hydrocarbon (1773 cm^−^^1^), and aldehyde (1179 cm^−^^1^), which are attributed to the volatilized products, were selected to be further investigated herein. The intensities of absorbance were normalized to the mass of the sample. By comparing Figure 7a with Figure 7b, the sample BAEA released much more CO, hydrocarbon, and aldehyde than the sample BAEA/DAPP 50/50, indicating the latter material provides the flame retardancy. The flame retardancy of DAPP is therefore confirmed.

**Figure 8:**
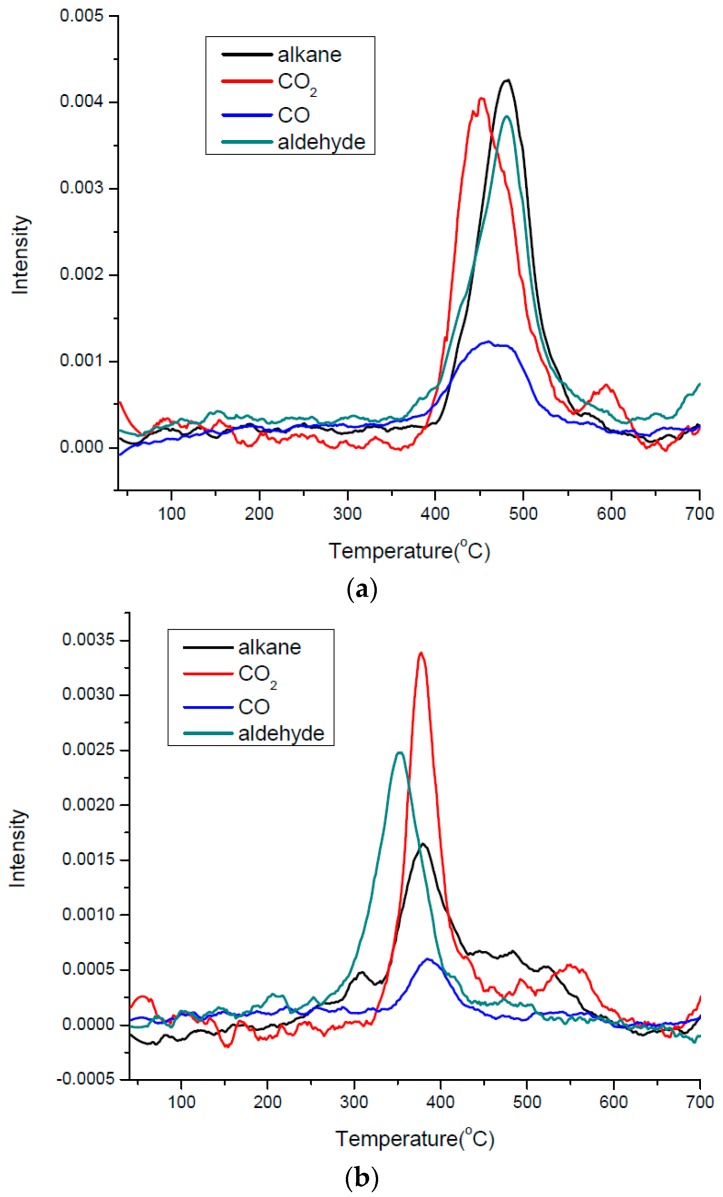
Intensity of evolved products, including hydrocarbon, CO_2_, CO, and aldehyde, against temperature in the pyrolysis of (**a**) pure DAPP or (**b**) DAPP/BAEA 30 mol %.

**Figure 9:**
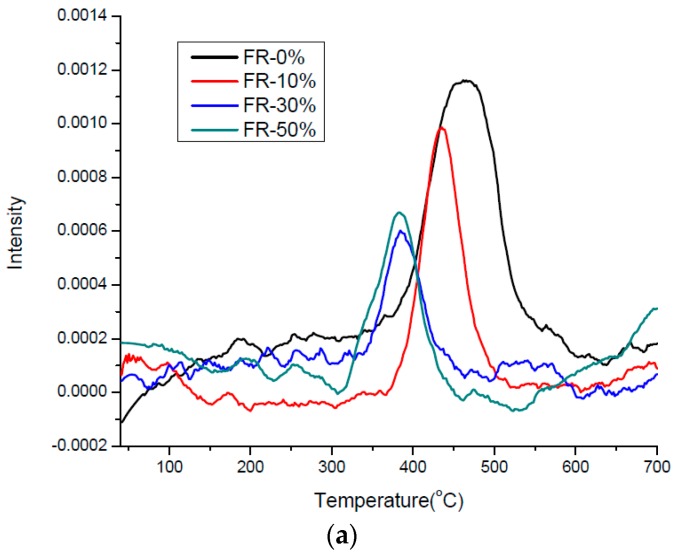

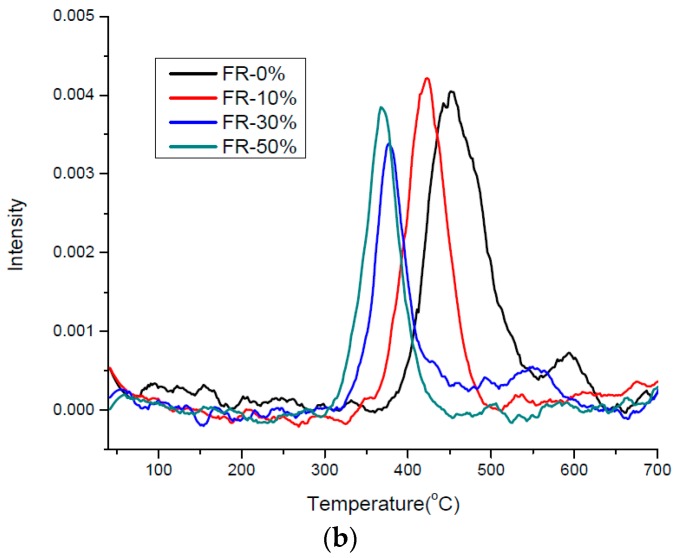
The intensity of evolved products for (**a**) CO and (**b**) CO_2_ in the pyrolysis of DAPP/BAEA with various ratios in a N_2_-rich environment.

With reference to the temperature scanning obtained by TGA instrument, the time scale in Figure 7 for a constant heating rate of 10 °C/min can be converted to a temperature scale, as shown in Figure 8. Figure 8a,b show plots of the intensity of the evolved gas versus temperatures for 0% and 30 mol % DAPP, respectively. Figure 8b shows significantly less intense peaks for the released gases CO, hydrocarbon and aldehyde, than those in Figure 8a. For sample DAPP/BAEA 30 mol %, the intensity of the CO_2_ peak was 90% of its original intensity without DAPP and the peak position was shifted to a lower temperature by 70 °C from its original temperature without DAPP. Figure 9a,b show plots of the peak intensity of evolved gases CO and CO_2_ versus temperature, respectively, for BAEA/DAPP in various ratios. The results again demonstrate that the amount of released CO gas decreases significantly as the phosphorus content increases. In contrast, the peak intensity of CO_2_ remains constant and is independent of the phosphorus content, but the temperature at which CO_2_ is released is significantly lowered as the blended phosphorus content is increased. As indicated above, the easy decomposition of the phosphorus compound during the initial stage forms an insulating layer, which can inhibit further burning of the resin and the consequent release of other flammable gases. Furthermore, CO_2_ is nonflammable and thus can dilute the concentration of combustible products and oxygen to obstruct combustion of materials during the decomposition of the phosphorus-containing compound. The initial decomposition releases a very large amount of CO_2_, which inhibits further burning. Briefly, DAPP reduces the release of combustible products and increases the yield of nonflammable products to improve the flame retardancy of polymeric materials to which it is added.

The cone-calorimeter is the most effective device for elucidating the combustion behavior of organic materials. The main information that is obtained using such an instrument is the HRR (Heat Release Rate) against time. Usually, the maximum release rate, HRR (Peak of Heat Release Rate), is taken to represent the burning behavior. The THE (Total Heat released at End) is the integral of the HRR (Heat Release Rate) from time zero to the end of the test, and represents the total heat output in the burning process. THE can be further divided by the total mass of the sample to obtain the EHC (Average Effective Heat of Combustion). Table 3 shows that BAEA samples that contains more phosphorus content from a DAPP compound have lower HRR, THE, and EHC than pure BAEA. All of the samples that contained DAPP herein had an HRR of less than 200 kW/m^2^, reduced from 378 kW/m^2^, confirming flame retardancy. The parameters are negligibly higher than those of FR-30% to FR-50%, indicating that the compounding of 30 mol % DAPP into BAEA is optimal for flame retardancy and the suppression of combustion. 

### 3.4. Kinetics of Pyrolysis

**Figure 10:**
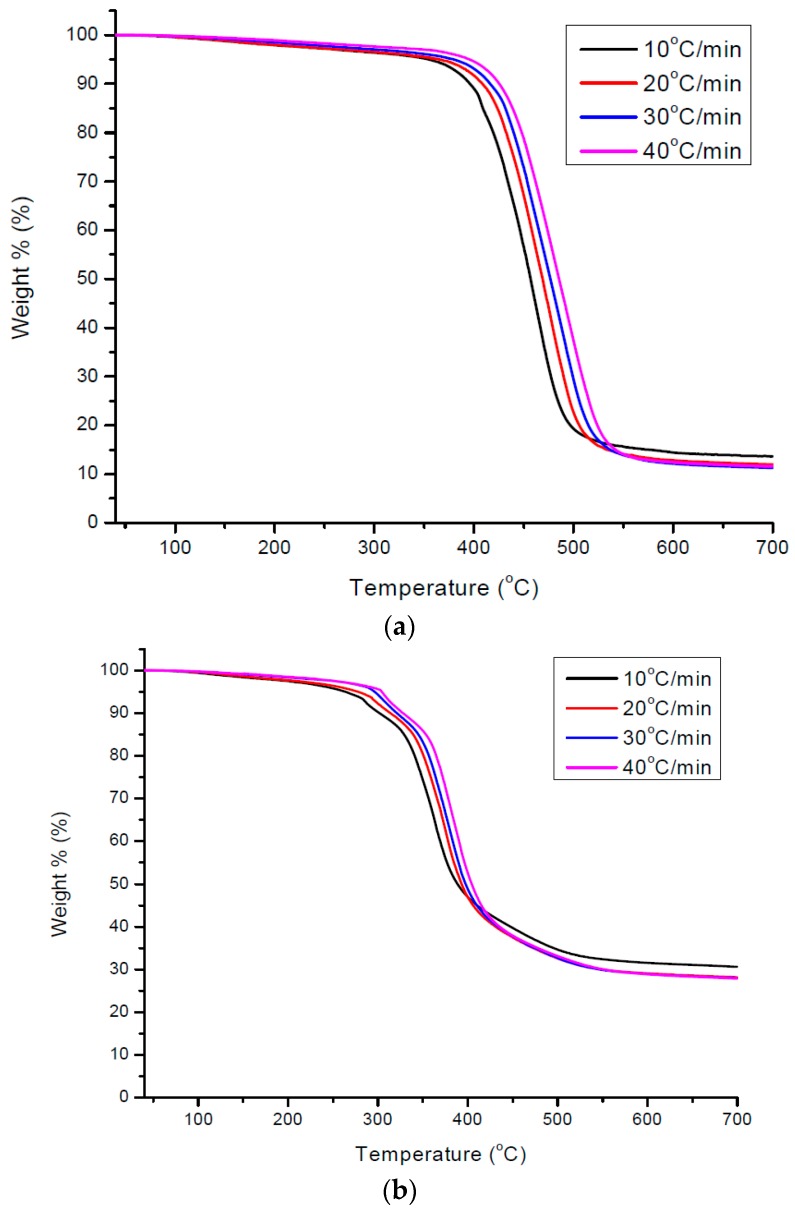
Typical TGA spectra at various heating rates in N_2_ for cured samples of (**a**) pure DAPP or (**b**) DAPP/BAEA 30 mol %.

The thermo-chemical decomposition behavior of BAEA/DAPP systems can be measured to examine the kinetics of their pyrolysis using the TGA instrument. The activation energy, *E_a_*, of the degradation of BAEA or BAEA/DAPP systems can be determined by Ozawa’s method [29,30], according to Equation (2), which estimates the *E_a_* of a given weight loss from the TGA spectra at various heating rates (Figure 10a,b). The average activation energy can then be calculated by averaging out all *E_a_* values for each conversion ratio:

*E_a_* (kJ/mole) = 2.19 × R × d[log(β)]/d[1/*T*] (2)

where β is the heating rate, *T* is the selected temperature, and R is the gas constant.

**Figure 11:**
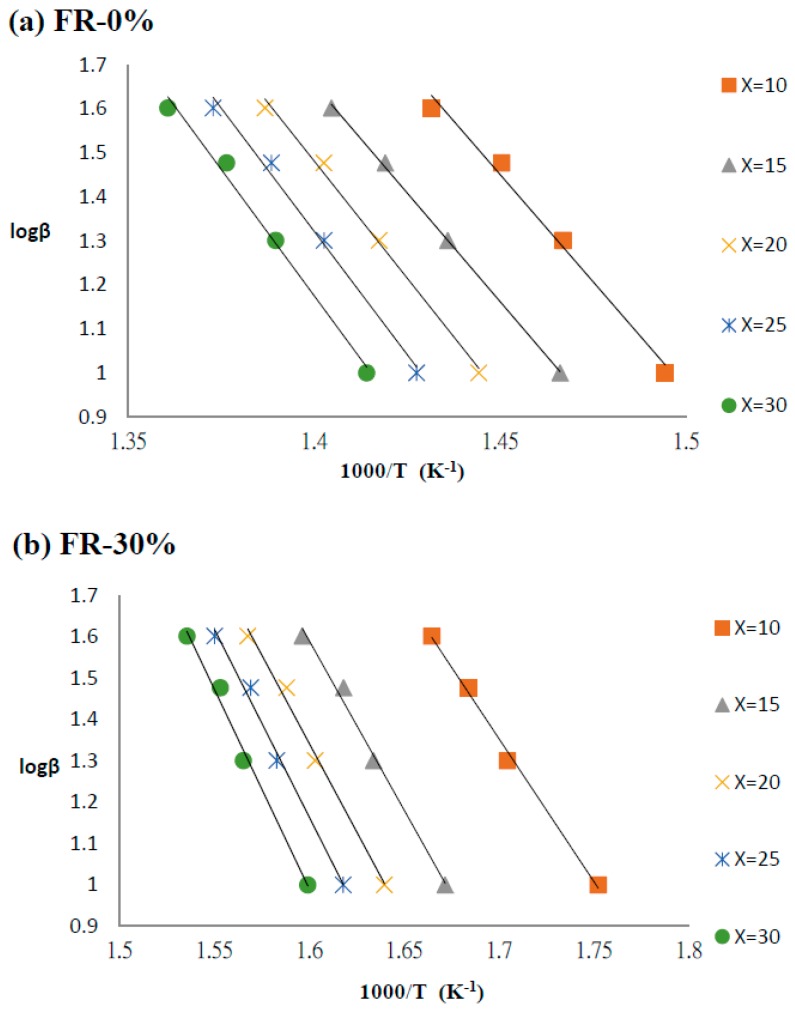
The typical treatment of thermogravimetric analysis traces for (**a**) pure DAPP and (**b**) DAPP/BAEA 30 mol %, by Ozawa’s method, to obtain the activation energy of degradation at various conversion ratios. The *E_a_* values are tabulated in Table 4.

**Figure 12:**
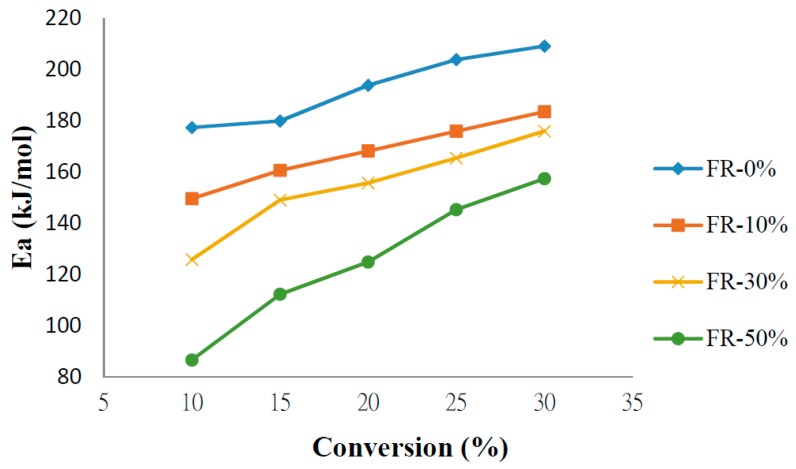
*E_a_* of degradation against conversion (%) for DAPP/BAEA mixture in four different molar ratios (0/100, 10/90, 30/70, and 50/50).

Figure 11a,b reveal a good linear relationship between log(β) and 1/*T* for BAEA and BAEA/DAPP (70/30), respectively, at conversion ratios from 10% to 30%. This finding demonstrates that single activation energy can be applied to depict the thermal decomposition of the BAEA/DAPP system. The mean *E_a_* values for the degradation of BAEA/DAPP in ratios of 10 and 50 mol % were similarly calculated, plotted in Figure 12, and detailed in Table 4. Figure 12 and Table 4 reveal that the BAEA/DAPP (50/50) system has the lowest *E_a_* of degradation, indicating that this system decomposes most easily. Experimental results again demonstrate that the mean *E_a_* decreases with the phosphorus content, owing to the ease of decomposition of the phosphorus-containing compound during the initial stage to form an insulating layer. This explanation is supported by the variation of *T_d_*, presented previously.

## 4. Conclusions

A UV curable flame retardant monomer DAPP that is based on BPDCP and HEA was synthesized and characterized by ^1^H NMR, ^31^P NMR, and IR spectrophotometer. The DAPP was then blended with regular BAEA at various blend ratios to yield various phosphorus contents. Experimental results show that the optimal concentration of photoinitiator is approximately 5 wt %, at which concentration both the reaction rate constant *k* and the final conversion α are maximal. The TGA-IR results demonstrate that compounding 30 mol % DAPP into BAEA significantly reduced the amount of released CO gas. In contrast, the peak intensity of CO_2_ is independent of phosphorus content. The LOI, reaching the saturated value of 26, and the HRR measured using a cone-calorimeter, 156.43 KW/m^2^, confirm the saturation point when 30 mol % DAPP was compounded into BAEA. The results from the cone-calorimeter reveal that BAEA samples blended with more phosphorus content from a DAPP compound have lower HRR, THE, and EHC than pure BAEA. A study of the kinetics of pyrolysis reveals that *E_a_* decreases as the phosphorus content increases. Finally, all the results shown above indicate that the phosphorus compound DAPP is easily decomposed during the initial stage of burning to form an insulating layer, which inhibits further burning of the resin and the consequent release of other flammable gases.

## Figures and Tables

**Table 1:** The fitting parameters obtained from PDSC for DAPP cured with various amounts of photoinitiator.

Photoinitiator	Δ*H* (J/g)	*k* (L/min)	*m*	*n*	Conversion (α)
1 wt %	216.51	13.51	0.70	1.62	54.85
3 wt %	229.10	16.41	0.70	1.64	57.47
5 wt %	232.85	18.61	0.70	1.63	60.41
7 wt %	224.97	14.24	0.70	1.65	56.43

**Table 2:** The results from TGA (thermogravimetric analysis) and LOI (limiting oxygen index) for DAPP/BAEA mixture with different ratios.

Sample	P (wt %)	Nitrogen Atmosphere			Air Atmosphere		
		*T_g_* (°C)	*T_d_* (°C)	700 °C Residue (wt %)	*T_d_* (°C)	700 °C Residue (wt %)	LOI
FR-0%	0	72.52	380.16	11.58	370.87	0.35	22.0 ± 0.5
FR-10%	0.72	69.72	311.28	18.63	335.73	3.60	23.0 ± 0.5
FR-20%	1.44	69.00	299.02	27.57	301.61	17.34	24.5 ± 0.5
FR-30%	2.15	62.00	286.01	28.20	297.77	21.76	26.0 ± 1
FR-40%	2.87	52.67	282.68	29.62	296.49	23.85	26.5 ± 1
FR-50%	3.59	51.57	271.02	29.82	284.35	27.09	26.5 ± 1

**Table 3:** The burning factors obtained from cone-calorimeter measurement for DAPP/BAEA in various ratios.

Sample	Phosphorus (wt %)	HRR (kw/m^2^)	THE (MJ/m^2^)	EHC (MJ/m^2^)	SEA (m^2^/kg)
FR-0%	0	378.88	54.86	29.77	1203.58
FR-10%	0.72	175.89	48.73	28.75	619.35
FR-30%	2.15	156.43	34.50	26.76	471.91
FR-50%	3.59	155.25	33.58	25.01	414.65

**Table 4:** The *E_a_* of degradation at different conversions for DAPP/BAEA mixture blended in various ratios.

	Sample	FR-0%	FR-10%	FR-30%	FR-50%
Conversion Ratio					
*X* = 10		177.4291	149.6557	125.8781	86.66933
*X* = 15		179.9557	160.6835	149.1551	112.3091
*X* = 20		193.9051	168.2453	155.7831	124.9224
*X* = 25		203.9353	175.9927	165.5293	145.3633
*X* = 30		209.2144	183.6201	176.0255	157.4742
Average		192.89 ± 16.33	167.64 ± 17.98	154.47 ± 21.56	125.3477 ± 32.12

## References

[1] Chang S.J., Chang F.C. (1998). Characterizations for blends of phosphorus-containing copolyester with poly(ethylene terephthalate). Polym. Eng. Sci..

[2] Huang Z., Shi W. (2007). Thermal degradation behavior of hyperbranched polyphosphate acrylate/tri (acryloyloxyethyl) phosphate as an intumescent flame retardant system. Polym. Degrad. Stab..

[3] Chen X., Hu Y., Jiao C., Song L. (2007). Thermal and UV-curing behavior of phosphate diacrylate used for flame retardant coatings. Prog. Org. Coat..

[4] Huang Z., Shi W. (2006). Synthesis and properties of poly(bisphenol A acryloxyethyl phosphate) as a UV curable flame retardant oligomer. Eur. Polym. J..

[5] Huang Z., Shi W. (2007). UV curing behavior of hyperbranched polyphosphate acrylate/di(hydroxylpropyl methacrylate) piperazine and properties of the cured film. Prog. Org. Coat..

[6] Xing W., Hu Y., Song L., Chen X., Zhang P., Ni J. (2009). Thermal degradation and combustion of a novel UV curable coating containing phosphorus. Polym. Degrad. Stab..

[7] Zhu S.W., Shi W.F. (2002). Synthesis and photopolymerization of hyperbranched polyurethane acrylates applied to UV curable flame retardant coatings. Polym. Int..

[8] Liang H., Asif A., Shi W. (2005). Photopolymerization and thermal behavior of phosphate diacrylate and triacrylate used as reactive-type flame-retardant monomers in ultraviolet-curable resins. J. Appl. Polym. Sci..

[9] Liang H., Asif A., Shi W. (2005). Thermal degradation and flame retardancy of a novel methacrylated phenolic melamine used for UV curable flame retardant coatings. Polym. Degrad. Stab..

[10] Cheng X., Shi W. (2010). UV-curing behavior and properties of tri/di (acryloyloxyethyloxy) phenyl silane used for flame-retardant coatings. Prog. Org. Coat..

[11] Huang Z., Shi W. (2006). Thermal behavior and degradation mechanism of poly(bisphenyl acryloxyethyl phosphate) as a UV curable flame-retardant oligomer. Polym. Degrad. Stab..

[12] Xing W., Song L., Jie G., Wang X. (2010). Preparation, flame retardancy and thermal behavior of a novel UV-curable coating containing phosphorus and nitrogen. Mater. Chem. Phys..

[13] Wang X., Wang B., Xing W., Tang G., Zhan J., Yang W. (2014). Flame retardancy and thermal property of novel UV-curable epoxy acrylate coatings modified by melamine-based hyperbranched polyphosphonate acrylate. Prog. Org. Coat..

[14] Cook W.D. (1992). Thermal aspects of the kinetics of dimethacrylate photopolymerization. Polymer.

[15] Young J.S., Bowman C.N. (1999). Effect of Polymerization Temperature and Cross-Linker Concentration on Reaction Diffusion Controlled Termination. Macromolecules.

[16] Cook W.D. (1993). Photopolymerization kinetics of oligo(ethylene oxide) and oligo(methylene) oxide dimethacrylates. J. Polym. Sci. Part. A Polym. Chem..

[17] Rwei S.P., Chen J.D., Su C.M. (2013). Kinetics of UV-curing of waterborne polyurethane acrylate dendrimer. Polym. Bull..

[18] Lindholm J., Brink A., Hupa M. (2009). Cone Calorimeter—A Tool for Measuring Heat Release Rate.

[19] Schartel B., Hull T.R. (2007). Development of fire-retarded materials—Interpretation of cone calorimeter data. Fire Mater..

[20] Huggett C. (1980). Estimation of rate of heat release by means of oxygen consumption measurements. Fire Mater..

[21] (2013). Method of Test for Heat Release Rate for Building Materials—Part 1: Cone Calorimeter Method.

[22] Nair C.P.R., Clouet G., Guilbert Y. (1989). Flame and thermal resistance of phosphorus-functionalized poly(methyl methacrylate) and polystyrene. Polym. Degrad. Stab..

[23] Kamal M.R., Sourour S. (1973). Kinetics and thermal characterization of thermoset cure. Polym. Eng. Sci..

[24] Kamal M.R. (1974). Thermoset characterization for moldability analysis. Polym. Eng. Sci..

[25] Sourour S., Kamal M.R. (1976). Differential scanning calorimetry of epoxy cure: Isothermal cure kinetics. Thermochim. Acta.

[26] Schartel B B. (2010). Phosphorus-based flame retardancy mechanisms—Old hat or a starting point for future development?. Materials.

[27] Van Krevelen D.W. (1977). FLAME RESISTANCE OF CHEMICAL FIBERS. Appl. Polym. Symp..

[28] Rwei S.P., Cheng C.Y., Liou G.S., Cheng K.C. (2005). Curing and pyrolysis of cresol novolac epoxy resins containing BABODOPN. Polym. Eng. Sci..

[29] Rwei S.P., Kao S.C., Liou G.S., Cheng K.C., Guo W. (2003). Curing and pyrolysis of epoxy resins containing 2-(6-oxido-6H-dibenz(c,e)(1,2)oxaphosphorin-6-yl)-1,4-naphthalenediol or bisphenol S. Colloid Polym. Sci..

[30] Rwei S.P., Liu A.Y., Liou G.S., Cheng K.C., Guo W. (2004). Curing and Pyrolysis of Epoxy Resins Containing ODOPN [2-(6-oxido-6H-dibenz(c,e) (1,2) oxaphosphorin-6-yl)-1,4-naphthalenediol] or Bisphenol-S. Polym. Eng. Sci..

